# Supplementary material for: Antibiofilm and anti-quorum sensing activity of Psidium guajava L. leaf extract: In vitro and in silico approach
Source: PLoS One. 2023 Dec 19;18(12):e0295524. doi: 10.1371/journal.pone.0295524 (PMC10729950; doi:10.1371/journal.pone.0295524)
Supplement: S1 File — (DOCX) [file pone.0295524.s001.docx]

**Antibiofilm and anti-quorum sensing activity of *Psidium guajava* L. leaf extract: *in vitro* and *in silico* approach**

Mo Ahamad Khan^a^, Ismail Celik^b^, Sachin Kumar^a^, Anwar Shahzad^c^, Haris M. Khan^a^, Mohammad Shahid^d^_,_ Bilal Ahmed^e^*****

1. Department of Microbiology, Faculty of Medicine, Aligarh Muslim University, Aligarh, 202002, India
2. Department of Pharmaceutical Chemistry, Faculty of Pharmacy, Erciyes University, 38000 Kayseri, Turkey
3. Department of Botany, Faculty of Life Science, Aligarh Muslim University, Aligarh, 202002, India
4. Department of Microbiology, Immunology and Infectious Diseases, College of Medicine and Medical Science, Arabian Gulf University, 26671, Kingdom of Bahrain
5. *Agricultural and Biological Engineering, Purdue University, West Lafayette, 47907, IN, USA

Corresponding author: Bilal Ahmed (ahmed249@purdue.edu)

**Supporting Information Captions**

**Table S1**. Inhibition of virulence factors and biofilm in *P. aeruginosa* by sub-MICs of PGME

**Table S2.** Phytocompounds of PGME as identified through GC–MS analysis.

**Table S3**. Autodock Vina molecular docking binding energy results (kcal/mol) of compounds alpha-Copaene, beta-Caryophyllene and Nerolidol against selected target proteins **CviR** (PDB ID: 3QP1), **LasR** (PDB ID: 2UV0), **LasI** (PDB ID: 1RO5) and **RhlR** (UniProtKB: P54292)

**Figure S1.** (A) Predicted 3D structure obtained from AlphaFold protein structure database and (B) Ramachandran plot of *P. aeruginosa* regulatory protein RhlR

**Figure S2.** (A) Superimposes of cocrystal ligands (gray) and redocked ligands (green) of CviR (PDB ID: 3QP1), and (B) LasR (PDB ID: 2UV0). RMSD values are 0.62 Å and 0.78 Å, respectively.

### **Table S1**. Inhibition of virulence factors and biofilm of *P. aeruginosa* by sub-MICs of PGME

| PGME concentration | Pyocyanin | Pyoverdin | Rhamopholipid | Biofilm | EPS |
| --- | --- | --- | --- | --- | --- |
| Control | 7.12±0.27 | 940±29.1 | 1.09±0.06 | 1.27±0.10 | \| 1.313±0.02 \| \| --- \| |
| DMSO (0.5%) | 7.15±0.29 | 937±25.2 | 1.05±0.03 | 1.20±0.13 | 1.290±0.02 |
| MIC/2 | 2.09±0.05 | 341±20.1 | 0.38±0.05 | 0.38±0.01 | 0.416±0.01 |
| MIC/4 | 4.11±0.10 | 536±15.2 | 0.67±0.04 | 0.64±0.03 | 0.548±0.01 |
| MIC/8 | 5.21±0.11 | 657±25.2 | 0.78±0.02 | 0.83±0.02 | 0.715±0.01 |
| MIC/16 | 6.40±0.22 | 800±26.8 | 0.88±0.01 | 0.97±0.02 | 0.879±0.02 |

Pyocyanin concentrations were expressed as µg/ml of culture supernatant; Pyoverdin results are expressed in fluorescence intensity (arbitrary unit); Total protease activity is expressed as the absorbance at 400 nm; Rhamnolipid is expressed as the absorbance at 421 nm; EPS production is expressed as the absorbance 490 nm. The data represent mean values of three independent experiments. ***** indicates p ≤0.05 and ****** indicates p ≤ 0.01 with respect to control.

### **Table S2.** Phytocompounds of PGME as identified through GC–MS analysis.

| S No. | R.Time | Area% | Name |
| --- | --- | --- | --- |
| 1 | 4.660 | 0.18 | [Methyl 2-methylbutyrate](https://www.ncbi.nlm.nih.gov/pcsubstance/?term=%22Methyl%202-methylbutyrate%22%5bCompleteSynonym%5d%20AND%2013357%5bStandardizedCID%5d) |
| 2 | 5.339 | 0.47 | tripinyl acetate |
| 3 | 5.548 | 17.60 | Alpha-copaene |
| 4 | 5.981 | 2.37 | Acetic acid, pentyl ester |
| 5 | 6.199 | 27.96 | Caryophyllene |
| 6 | 6.22 | 1.05 | 5-(Hydroxymethyl)-2-(dimethoxymethyl)furan |
| 7 | 6.613 | 0.32 | Bicyclo[5.3.0]decane, 2-methylene-5-(1-methylvinyl)-8-methyl- |
| 8 | 7.160 | 0.32 | D-Limonene |
| 9 | 7.313 | 0.51 | Acetic acid, nonyl ester |
| 10 | 7.616 | 1.49 | 1,2-Cyclohexanedicarboxylic acid, ethyl 4-methoxyphenyl |
| 11 | 8.920 | 0.21 | Citronella |
| 12 | 9.537 | 22.30 | Nerolidol |
| 13 | 10.810 | 3.58 | 1,6-Anhydro-.alpha.-d-galactofuranose |
| 14 | 11.018 | 5.52 | Bisabolene |
| 15 | 12.375 | 0.43 | Hexanoic acid, 2,6-dimethylnon-1-en-3-yn-5-yl ester |
| 16 | 12.901 | 0.84 | 2-Furancarboxylic acid, 3-methyl-, methyl ester |
| 17 | 13.042 | 1.88 | Quercetin |
| 18 | 13.484 | 2.25 | Alloaromadendrene |
| 19 | 13.697 | 0.38 | Carrophyllene oxide |
| 20 | 13.864 | 0.33 | Dodecamethyl cyclohexasiloxane |
| 21 | 14.150 | 0.49 | Heptadecanoic acid, ethyl ester |
| 22 | 14.431 | 0.36 | Propanoic acid, 3-mercapto-, dodecyl ester |
| 23 | 15.116 | 2.41 | 9,12-Octadecadienoic acid (Z,Z)-, methyl ester |
| 24 | 15.178 | 2.26 | 6-Octadecenoic acid, methyl ester, (Z)- |
| 25 | 15.412 | 0.32 | Alpha-cadinene |
| 26 | 15.502 | 0.47 | 1-(Dichloromethyl)dimethylsilyloxy-3-methylbut-2-ene |
| 27 | 16.954 | 0.34 | Hexadeca-2,6,10-tetraen |
| 28 | 19.358 | 1.01 | 1,2-benzenedicarboxylic acid |
| 29 | 21.715 | 2.35 | 9-octadecenamide |
|  |  | 100.00 |  |

**Table S3**. Autodock Vina molecular docking binding energy results (kcal/mol) of compounds alpha-Copaene, beta-Caryophyllene and Nerolidol against selected target proteins **CviR** (PDB ID: 3QP1), **LasR** (PDB ID: 2UV0), **LasI** (PDB ID: 1RO5) and **RhlR** (UniProtKB: P54292)

|  | **Target proteins** | | | |
| --- | --- | --- | --- | --- |
| **Compounds** | **CviR**  (PDB: 3QP1) | **LasR**  (PDB ID: 2UV0) | **LasI**  (PDB ID: 1RO5) | **RhlR**  (UniProtKB: P54292) |
| alpha-Copaene | -6.6 | -7.9 | -6.6 | -6.4 |
| beta-Caryophyllene | -7.0 | -8.6 | -6.5 | -6.5 |
| Nerolidol | -7.6 | -8.2 | -5.7 | -5.5 |


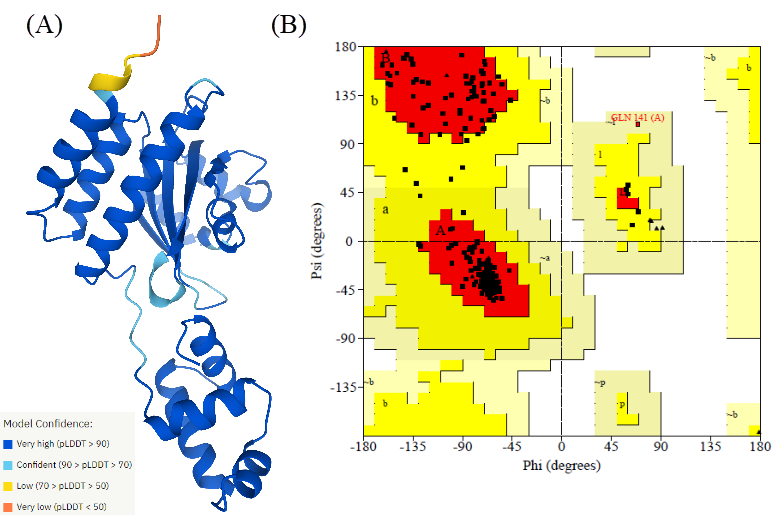


### **Figure S1.** (A) Predicted 3D structure obtained from AlphaFold protein structure database and (B) Ramachandran plot of *Pseudomonas aeruginosa* regulatory protein RhlR


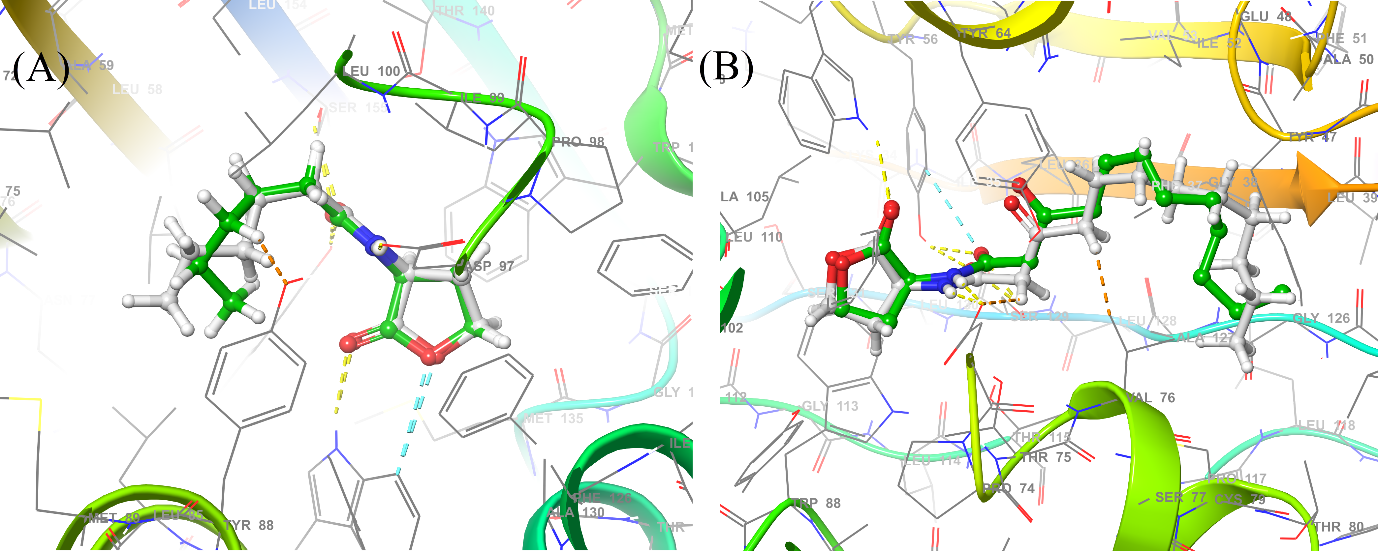
**Figure S2.** (A) Superimposes of cocrystal ligands (gray) and redocked ligands (green) of CviR (PDB ID: 3QP1), and (B) LasR (PDB ID: 2UV0). RMSD values are 0.62 Å and 0.78 Å, respectively.
