# Supplementary material for: Antibiofilm and anti-quorum sensing activity of Psidium guajava L. leaf extract: In vitro and in silico approach
Source: PLoS One. 2023 Dec 19;18(12):e0295524. doi: 10.1371/journal.pone.0295524 (PMC10729950; doi:10.1371/journal.pone.0295524)
Supplement: Attachment — Submitted filename: Response to Reviewers.docx [file pone.0295524.s002.docx]

**Response to Editor’s comments:**

When submitting your revision, we need you to address these additional requirements.

1. Please ensure that your manuscript meets PLOS ONE's style requirements, including those for file naming. The PLOS ONE style templates can be found at

https://journals.plos.org/plosone/s/file?id=wjVg/PLOSOne_formatting_sample_main_body.pdf and

https://journals.plos.org/plosone/s/file?id=ba62/PLOSOne_formatting_sample_title_authors_affiliations.pdf

**Response-** The manuscript matches Plos One formatting style requirements.

2. We suggest you thoroughly copyedit your manuscript for language usage, spelling, and grammar. If you do not know anyone who can help you do this, you may wish to consider employing a professional scientific editing service.

Upon resubmission, please provide the following:

● The name of the colleague or the details of the professional service that edited your manuscript

● A copy of your manuscript showing your changes by either highlighting them or using track changes (uploaded as a *supporting information* file)

● A clean copy of the edited manuscript (uploaded as the new *manuscript* file)

**Response-** The language of the manuscript has been copyedited by Bilal Ahmed, Purdue University, USA and reviewed by a colleague.

3. Thank you for stating the following financial disclosure:

"No"

At this time, please address the following queries:

a) Please clarify the sources of funding (financial or material support) for your study. List the grants or organizations that supported your study, including funding received from your institution.

b) State what role the funders took in the study. If the funders had no role in your study, please state: “The funders had no role in study design, data collection and analysis, decision to publish, or preparation of the manuscript.”

c) If any authors received a salary from any of your funders, please state which authors and which funders.

d) If you did not receive any funding for this study, please state: “The authors received no specific funding for this work.”

Please include your amended statements within your cover letter; we will change the online submission form on your behalf.

**Response-** We have addressed the queries in the revised manuscript as:

**Funding:** The corresponding author Bilal Ahmed is employed by Purdue University, USA. Authors Mo Ahmad Khan, Anwar Shahzad, Haris M. Khan are employed by Aligarh Muslim University, India. Ismail Celik is employed by Erciyes University, Turkey and Mohammad Shahid is currently employed by Arabian Gulf University, Kingdom of Bahrain. The authors received no specific funding for this work. The funders had no role in study design, data collection and analysis, decision to publish, or preparation of the manuscript.

4. PLOS requires an ORCID iD for the corresponding author in Editorial Manager on papers submitted after December 6th, 2016. Please ensure that you have an ORCID iD and that it is validated in Editorial Manager. To do this, go to ‘Update my Information’ (in the upper left-hand corner of the main menu), and click on the Fetch/Validate link next to the ORCID field. This will take you to the ORCID site and allow you to create a new iD or authenticate a pre-existing iD in Editorial Manager. Please see the following video for instructions on linking an ORCID iD to your Editorial Manager account: https://www.youtube.com/watch?v=_xcclfuvtxQ

**Response-** The ORCID ID has been mentioned in the Editorial Manager.

5. Please include captions for your Supporting Information files at the end of your manuscript, and update any in-text citations to match accordingly. Please see our Supporting Information guidelines for more information: http://journals.plos.org/plosone/s/supporting-information.

**Response-** Captions for Supporting Information are added at the end of the manuscript.

Additional Editor Comments:

The work presented to Plos One is part of the search for new control strategies for pathogens, different from the use of antibiotics. In this case, inhibiting the deployment of virulence systems by inhibiting bacterial quorum sensing. Although the work presented is interesting and logical in its structure, it is possible to appreciate details framed in the field of microbiology related to experimental design, methodologies, expression of results and their interpretation, which cast doubt on the scientific conclusions obtained. The English language must be revised.

In the work presented, extracts of natural origin of the species Psidium guajava are evaluated to be evaluated against the bacterium Pseudomonas aeruginosa and its virulence mechanisms associated with pathogenicity. The authors design experiences with biosensors for use in this area such as C. violaceum and evaluate the effect of the extracts on the expression of virulence factors such as the expression of EPS, rhamnolipids.

**Response-** We have thoroughly improved the English language of the manuscript along with methodology, expression of results and the discussion. Each section has been revised to weed out all sorts of ambiguity and errors. Methodologies have been elaborated, results and discussion have been edited to bring more clarity. Grammatical and typo errors have been rectified.

Additionally, depending on the chemical characterization of the active extracts, the authors carry out the exercise of modeling the possible molecular interactions, which is interesting, however, it is not conclusive due to the following critical precedents found in the article:

1. Line 132, method to quantify violacein. The authors state: "Following incubation, the treated culture was centrifuged to isolate the insoluble violacein, which was then suspended in DMSO and centrifuged again to remove the cells. The resulting supernatants were then transferred to microtiter plate wells for the measurement of violacein at 585 nm." Unfortunately, although the methodology is precise in the method reference, there is no detailed experimental protocol that allows determining the relationship between violacein production and cell number or biomass. this is critical and necessary in terms of properly normalizing the data.

**Response-** Thank you for pointing out this comment. We have modified the methodology for quantification of violacein.

2. A similar situation can be observed in the methodology in lines 136-146 in which the Biofilm Inhibition assay method is described. There is no relationship between the measurement of crystal violet color with respect to a relative or normalized value with the bacterial biomass in culture or number of cells.

**Response**- A biofilm inhibition experiment was conducted at sub-MIC levels. At the sub-MIC level, there was no effect on bacterial cell number. The bacteria produced biofilms which was surrounded by a matrix of extracellular polymeric substances (EPS). This EPS attached to the outer edge of each well where the plate, media, and air intersect. This can be observed as a thin, dark purple ring after staining. The amount of crystal violet absorbed by the bacteria is proportional to the number of cells in the biofilm. The treated wells produced a lower amount of EPS or biofilm compared to the control, as mentioned above, where sub-MIC levels did not affect the bacterial cell numbers.

3. According to the above, there is a problem related to the data used to conclude the effect of an extract, its characterization and subsequent molecular interaction tests, which may be unconsciously wrong due to a methodological failure. For these reasons, it is necessary for the authors to review their manuscript and establish whether the results expressed in graphs 2, 3 and 5 are correct or do not represent reality once the data has been normalized with the records of bacterial biomass or number of cells. (turbidity or CFU).

**Response**- All the experiments were carried out at sub-MICs of the extract. Growth kinetics studies were performed to confirm that there was not significant effect of the extract on the growth of the test pathogens. Growth curves of treated and untreated is provided in the revised manuscript. We did not normalize any data in the manuscript.

Log CFU/ml

| Time (h) | 0-MIC | 1/2-MIC |
| --- | --- | --- |
| 1 | 6.22 | 6.12 |
| 2 | 7.22 | 6.99 |
| 3 | 8.71 | 8.21 |
| 4 | 9.53 | 9.19 |
| 5 | 10.91 | 10.26 |

Log CFU/ml

| Time (h) | 0-MIC | 1/2-MIC |
| --- | --- | --- |
| 1 | 5.82 | 5.61 |
| 2 | 6.63 | 6.14 |
| 3 | 7.44 | 7.29 |
| 4 | 8.27 | 8.21 |
| 5 | 9.28 | 8.96 |

*Chromobacterium violaceum*

*Pseudomonas aeruginosa*

**Response to reviewers**

The study by Khan and colleagues reports the anti-quorum sensing and antibiofilm activity of methanolic extract of Psidium guajava leaves. The authors also analyzed the extract's main phytochemicals by GC-MS and described potential interactions of three constituents with some quorum sensing molecules by molecular docking.

1. A potential adverse effect of antimicrobials is the selection of resistant microorganisms. Thus, the information that the widespread use of antimicrobials causes resistance should be modified.

**Response**- Thank you for pointing this out. We have revised the statement in manuscript.

2. Natural products derived from plants have been used as antimicrobials since ancient times. Researchers who have renewed interest in studying these products.

**Response**- True, we appreciate the knowledge of learned reviewer.

3. Virulence factors are essential for bacterial survival in certain situations. For example, during an infectious process. Biofilms are the most common mode of microbial growth in different environments.

**Response**- You're absolutely right! Virulence factors are crucial for bacterial survival during infections, and biofilms are indeed the predominant mode of microbial growth in various environments. Understanding these aspects is vital for developing effective strategies to combat bacterial infections and manage biofilm-related challenges.

4. According to the authors, one of the objectives of the study is to evaluate the effect of *P. guajava* extract on the formation of virulence factors. The methodology used is not adequate to meet this objective, since the inhibitory effect on the expression of the selected virulence factor was analyzed.

**Response-** We appreciate your comment. Based on your comment, we recognize that our initial objective did not align with the methodology used in the study. We have taken your suggestion into consideration and have revised our objective accordingly to better reflect the actual focus of our research. Your input is valuable in ensuring the accuracy and relevance of our study's objectives

5. If one of the objectives is to select substances/extracts with potential anti-quorum sensing or anti-biofilm activity, why was only the methanolic extract, which showed growth inhibition activity, selected? Have the other extracts been tested on biofilms?

**Response-** We appreciate the reviewer's comment and understand the need for further clarification on selection of particular extract. We selected the methanolic extract because it demonstrated a unique dual activity of inhibiting bacterial growth and disrupting quorum sensing, as evidenced by the inhibition of violacein production. Our primary objective was to identify substances with potential anti-quorum sensing properties that could complement their antimicrobial effects. PGME exhibited both of these desired activities, making it a promising candidate for further investigation. But all other extracts do not show any activity.

6. What quality controls of the tests: substance with known inhibitory effect in relation to the test performed with the extract? Microdilution test control bacteria for MIC determination?

**Response-** We appreciate the reviewer's inquiry about the quality controls of the test. In our study, we did incorporate controls for various purposes. Firstly, in all experiments, we included DMSO (0.5%) as a control because we utilized it as a solvent to dissolve our plant extract. Additionally, during the microdilution tests, we employed two control groups. The first was a blank control, consisting of the growth medium without any plant extract, to assess the baseline bacterial growth. The second control involved the use of DMSO (0.5%) to account for any potential effects of the solvent on bacterial growth. These controls were implemented to ensure the accuracy and reliability of our experimental results.

7. In the violacein quantification test, a numerical value is expected for the results, and not as optical density values.

**Response-** Thank you for your response. We have removed the optical density value from the result.

8. The cell density of the inoculum for the biofilm assay must be reported in order for the result to be reproduced.

**Response-** The relevant section has been revised as: The impact of sub-MICs of PGME on biofilm development was assessed using a 96-well plate [22]. Bacterial culture in the mid-log phase were used to prepare the inoculum for biofilm assays. This phase typically represents a balanced growth state, where bacteria are actively dividing but have not yet entered stationary phase. The CFU/ml at this stage ranged from 10^6^ to 10^7^ CFU/ml which were dispensed into the 96-well MTP with 150 µL of LB medium, containing sub-MIC levels of PGME or without, and incubated at 37°C for 24 h. After incubation, PBS was used to wash the MTP wells to eliminate unattached cells. Crystal violet solution (0.1% w/v) was then added to visualize the biofilm, which adhered to the EPS and bacterial cells within it. The unbound crystal violet was removed through rinsing, and ethanol was used to solubilize the biofilm bound CV. The absorbance was measured at OD_620_ using spectrophotometry.

9. Biofilms were treated with which extract concentration for electron microscopy analyses? The methodology needs to be better described.

**Response-** Biofilms were treated with MIC/2 for electron microscopy analyses. The methodology has been improved.

10. The authors describe the results and compare them with other results obtained with different plants. However, this comparison must be done carefully, taking into account the plant species, the type of extract and the main chemical constituents.

**Response-** We have taken your suggestions into careful consideration to improve the discussion.

11. The English language must be revised.

**Response-** The English language of the manuscript has been thoroughly revised.

12. Line 263: What does "aggerate-like structure" mean

**Response-** It suggests that, "aggregate-like structures" in biofilm SEM images represent regions where microbial cells are densely packed and held together by the extracellular polymeric substances, forming distinct clusters or clumps of cells within the biofilm.

13. Line 267: “EPS is a major virulence factors” please explain.

**Response-** EPS is one of the major virulence factors which are mainly involved in biofilm formation and establishing the infection in host tissue. It also prevents the penetration of antibiotics substance into the bacterial cell (Ganesh, P. S., & Rai, V. R. (2018). EPS is a critical component of biofilms, significantly contributes to the ability of bacteria to establish and maintain infections by enhancing their resistance to treatment and their ability to evade the host's defenses. In the context of the study, the decrease in EPS production by PGME is significant because it suggests a potential mechanism by which PGME inhibits biofilm development and, consequently, reduces bacterial virulence.
