# Supplementary material for: Antibiofilm and anti-quorum sensing activity of Psidium guajava L. leaf extract: In vitro and in silico approach
Source: PLoS One. 2023 Dec 19;18(12):e0295524. doi: 10.1371/journal.pone.0295524 (PMC10729950; doi:10.1371/journal.pone.0295524)
Supplement: Attachment — Submitted filename: PLos review.docx [file pone.0295524.s003.docx]

The current study evaluates the anti-QS and anti-virulence activities of guava leaves against Pseudomonas aeruginosa. The study is well designed and conclusive.

The mani concern: please show the effect on the bacterial growth at sub-MIC concentration

Minors:

- Please write the names of genes in italic
- Add more information about the advantages of employing this strategy in conquering bacterial resistance, please find helpful publications

<https://doi.org/10.3390/ijms232113088>

<https://doi.org/10.3390/microorganisms10101976>

<https://doi.org/10.3390/app11156847>

https://doi.org/10.3390/biomedicines10051169

- Furthermore, if there are previous studies exploring the guava leaves antibacterial and anti-virulence activities, please include in your results and discussion
