# Supplementary material for: Antibiofilm and anti-quorum sensing activity of Psidium guajava L. leaf extract: In vitro and in silico approach
Source: PLoS One. 2023 Dec 19;18(12):e0295524. doi: 10.1371/journal.pone.0295524 (PMC10729950; doi:10.1371/journal.pone.0295524)
Supplement: Attachment — Submitted filename: Response to comments.docx [file pone.0295524.s004.docx]

**Comment:** Line 125 “*Antimicrobial screening and minimal inhibitory concentration (MIC) determination*” The authors do not present methodology information that allows supporting the work with known concentrations or proportions of the evaluated PGME extract. The authors point out that serial dilutions of the extract were made without indicating whether it was from the original stock solution or a dilution of the stock solution. Considering that the work refers to the evaluation of an extract in its biological activities such as anti-virulence or antibiofilm, not having this information is critical for the quality of the paper.

**Response**- We have clarified the issue regarding the stock solution in the methodology. We have written the serial dilution concentration for MIC determination.

**Comment:** Line 131 The authors point out "to generate a concentration range". Again, not knowing the concentration of the extract of the initial stock solution or matrix is critical when indicating the "concentration range". The authors must indicate the original concentration of the extract or its percentage working ratio.

**Response**- The initial stock solution concentration was 8 mg/ml which has now been included in the methodology.

**Comment:** Line 139 “varying concentrations of the fractions were placed on the agar plate” The authors refer to a neither detailed nor defined concentration of a fraction of the extract containing molecules evaluated.

**Response-** Thank to point out this error. We have corrected it in methodology section.

**Comment:** Line 156 “Biofilm Inhibition Assay” The authors point out “containing sub-MIC levels of PGME”. The authors point out values that do not correspond to concentrations or proportions (percentages) that allow the MIC to be scientifically defined by concentration or proportion.

**Response-** The Sub-MIC concentration for *P. aeruginosa* ranged from 500-62.5 µg/ml (MIC/2-MIC/8) and 250-31.2 µg/ml (MIC/2-MIC/8) for *C. violaceum*.

**Reviewer #2**

1. **Comment:** The authors have adequately addressed the reviewer comments but the title should be modified and the introduction should be summarized.

**Response-** Thank you for your valuable feedback and suggestions. We have revised the title as per your recommendation. Additionally, we have summarized the introduction as suggested.

2. **Comment:** Also, Figure showing the inhibitory effect of the extract on the virulence factors should be added.

**Response**- Thank you for your suggestion. We have already included a figure, Figure 6, which demonstrates the inhibitory effect of the extract on the virulence factors.

3. **Comment:** More recent references should be cited like:

Alotaibi B, Negm WA, Elekhnawy E, El-Masry TA, Elseady WS, Saleh A, Alotaibi KN, El-Sherbeni SA. Antibacterial, immunomodulatory, and lung protective effects of Boswellia dalzielii oleoresin ethanol extract in pulmonary diseases: in vitro and in vivo studies. Antibiotics. 2021 Nov 25;10(12):1444.

Attallah NG, El-Kadem AH, Negm WA, Elekhnawy E, El-Masry TA, Elmongy EI, Altwaijry N, Alanazi AS, Al-Hamoud GA, Ragab AE. Promising antiviral activity of Agrimonia pilosa phytochemicals against severe acute respiratory syndrome coronavirus 2 supported with in vivo mice study. Pharmaceuticals. 2021 Dec 16;14(12):1313.

Attallah NG, El-Sherbeni SA, El-Kadem AH, Elekhnawy E, El-Masry TA, Elmongy EI, Altwaijry N, Negm WA. elucidation of the metabolite profile of Yucca gigantea and assessment of its cytotoxic, antimicrobial, and anti-inflammatory activities. Molecules. 2022 Feb 16;27(4):1329.

**Response**- Thank you for providing these references. We have included them in the revised manuscript.

**Reviewer #3:**

1. **Comment:** The main concern: please show the effect on the bacterial growth at sub-MIC concentration

**Response-** The time kill assay method was intended to show the effect of Sub-MIC of plant extract on bacterial cell which is now added in the revised manuscript.

2. **Comment:** Please write the names of genes in italic.

**Response-** Thank you for your feedback and suggestions. Changes are made to ensure the gene names italicized throughout.

3. **Comment:** Add more information about the advantages of employing this strategy in conquering bacterial resistance, please find helpful publications:

<https://doi.org/10.3390/ijms232113088>

<https://doi.org/10.3390/microorganisms10101976>

<https://doi.org/10.3390/app11156847>

<https://doi.org/10.3390/biomedicines10051169>

**Response-** Thank you for your valuable feedback and suggestions. We appreciate your comment regarding the need to provide more information about the advantages of the strategy in conquering bacterial resistance. We have cited these references.

4. **Comment:** Furthermore, if there are previous studies exploring the guava leaves antibacterial and anti-virulence activities, please include in your results and discussion

**Response-** Thank you for your valuable comment. We appreciate your suggestion regarding the inclusion of previous studies exploring antibacterial and anti-virulence activities of guava leaves in the results and discussion. Though there are very few studies of anti-virulence activities by guava extract. This is probably the first study which detailed about anti-virulence activity of guava leaf extract.
